# Democracy and Doctors

**Published:** 1852-08

**Authors:** R. H. Paddock

**Affiliations:** Cheshire, Ct.


From the Ohio Medical and Surgical Journal.

*Democracy and the Doctors.* By R. H. PADDOCK, M. D. Cheshire, Ct.

It is a commonly received opinion, that the old forms of government, being patriarchal in their character, and celestial in their origin, are favorable to the cultivation of the liberal arts and professions.

It is also just about as commonly supposed, that democracy can appreciate only that which is of obvious practical utility, and affords little countenance or protection to amateurs in literature or art, or to those engaged in the practice of the learned professions. A single individual, as a king, or a single and comparatively small class of persons, as an aristocracy,—having had the best of instructors and the most ample facilities for the acquisition of all useful and elegant accomplishments,—must surely better understand, and more safely guard the interests of learning and the learned; must more readily detect, and more profoundly abhor, all schemes of quackery and imposture, and will more effectually crush them, at a blow, than can be expected of a whole people, of all conditions—whether of fortune or misfortune. On the other hand, the very notion of liberty implies the toleration of manifest evils, and opens the road to eminence in all that is hateful and degrading, as well as in all that is lovely and exalting.

Now, we doctors see and feel so much of the *intolerable* impudence, conceit, and ignorance of *tolerated* quacks, that we are sometimes tempted to abjure our own political creed, and go back to the hated and antiquated forms of absolutism—seeking, under the iron wand of despotism, protection for ourselves, and vengeance on swaggering charlatanry. But alas! in this day to whom shall we go? To the leagued tyrants of continental Europe? All the beauties of absolutism, *in the abstract*, celestial in origin, as it *might be*, tender and parental, as it *should be*,—vanish at a glance of these miscreant ministers of an angry God! But, upon a "sober second thought," were despots less despotic, and tyranny even tolerable, we would not invoke its aid. Medical quackery has its cause and cure in something back of all forms of government; and, though sometimes not allowed to be practiced openly, it nevertheless insinuates itself into all States, however ruled or misruled.

A very common and very erroneous notion in reference to the nature of disease, furnishes those who have more brass than brains, with an opportunity to mislead a multitude to their own hurt. It is something like this: Every malady is distinct in its nature; well characterised; of specific form, and requiring a specific mode of treatment.

The doctor is expected to recognise diseases as readily and as certainly as the naturalist does plants and animals, and to apply to each the specific remedy which nature has provided. According to this simple and beautiful theory the practice of medicine should be a very easy and satisfactory business, and the doctor should be certain death, not only "on fits," but also on disease itself.

A good practical illustration of this notion is seen in that system of seething, spewing, and injecting, known as the Thomsonian. The semi-civilized discoverer of the wonderful secrets that heat is life and cold is death; that every substance should come out of the body through the same channel by which it enters it; and, that red pepper and lobelia, administered hot to both extremities of the body, will drive out the seeds of all disease, viz.: cold and canker—commenced his original treatise on the healing art by advising all men to shun the lawyers, the doctors, and the ministers of religion. The remainder of his book is a worthy commentary on the text, and like Joe Smith's Bible, finds those in every community, who are captivated by its vulgarity, and enlightened by its profound revelations.

There is something attractive, too, for a certain class of persons, in the operations of the Thomsonian doctor. His is no light duty—no easily earned reward! he throws off his coat, rolls up his sleeves, and swelters, for hours, over a steaming caldron of concocting boughs and herbs. The huge bowls of hot drinks, the bath frames covered with woolen blankets, and the red-hot, hissing stones, all show that something is to be done effectually, and that either the disease or the patient must yield before this formidable enginery.

Now, it is neither the highest nor the lowest portion of our race in point of intelligence, that is imposed upon by such theory and practice. Those nations which have undergone the process of calcination and calcitration, through the wickedness of the irrulers, till they can not, or dare not, aspire to think for themselves upon any subject, make the very best class of medical patients. They surrender their bodies to the legitimate doctors, and their souls to the lawful priests, to be healed of their respective maladies with an equally blind and unwavering faith in both. It is when the human mind has been released from the bondage of ignorance and oppression; when some straggling rays of light begin to fall, and it begins to put forth its early and uncertain efforts in speculative philosophy, that it embraces such crudities.

What wonder if, in the twilight of their mental illumination, when men have acquired just that "little learning," which is always a "dangerous thing," in speculating about doctoring themselves, as well as about governing themselves, and acting for themselves in every capacity—they should often adopt undigested schemes of corporeal, as well as of spiritual salvation? What wonder that their inexperienced ears should listen to the noisy quack, pasted all over with glaring certificates and lying advertisements?

Another form of medical imposture, more refined in its character, and perhaps quite as extensive in its influence as the preceding, owes its origin and advocacy to minds of a contemplative and visionary mould. These are found in the ranks of the most refined, intelligent and virtuous, and when controlled by a powerful judgment, as was that of Columbus, are often among the most distinguished of our race. Affluence and independence, or at least a condition of life exempt from the necessity for physical labor, are almost indispensable to the formation and cultivation of such a mental habit. The man who earns his daily bread by daily toil, has little time or inclination for day dreaming; while the student, the cultivators of science, art, and the learned professions, are quite liable to fall into speculating and theorizing.

Even the despicable tyrant, who enchains the bodies and minds of the great mass of his subjects, will sometimes foster the spirit of speculative philosophy, or of devotion to the fine arts. He is willing to engross the minds of the contemplative with such topics as can have no practical influence to enlighten or elevate the people, and for this reason he has sometimes been hailed as the patron of all liberal learning.

The medical hallucinations of these transcendental philosophers, like all their etherial lucubrations, are wonderfully exquisite and pseudo-logical. Their thread of ratiocination is microscopically attenuated, and their deductions are the doubly-refined extract of nonsense.

At one time they inform us that a wet sheet, wrapped around the human body, will certainly absorb the active elements of any disease; while a little Croton water, introduced within the body, is far more powerfully curative than Croton oil. At another time we are gravely told, that any cause which can derange the delicate mechanism of the human system, is itself the proper means of cure; and hence a sledge hammer is a fit tool to mend a broken watch; and also that the less is more powerful than the greater, of the same kind; and hence that nothing at all is absolutely omnipotent.

Furthermore—if any man doubts the truth of these startling propositions, they can be verified by thousands of testimonials, and by actual experiment. This can not be said of the propositions of Euclid—so away with your mathematics, and give us the documents!

Doubtless there are other forms of medical imposture, dependent upon other peculiarities in the constitution and condition of the human mind. These it is not my present purpose to exhibit, but rather to infer from what has already been said, that the sources and the remedies of charlatanry, are not to be sought in the forms of government; or in legislative enactments. Doubtless these have an important influence in the determination of mental conditions and characteristics; but men cannot be cured of ignorance or insanity, nor can they be endowed with a truly enlightened understanding, and good practical common sense, by the force of law. All experience shows that neither our moral nor our physical maladies are likely to be better healed when under the care of the State, than when left to the care and the conscience of the individual patients themselves.

Much then, as we are scandalized by the wide-spread medical quackery of our time and country—much as we deplore its soils, abhor its impudence, and despise its flimsy sophistry—still, we shall do well to adhere to our democratic notions of government—giving the fool full liberty to preach folly, and his hearers abundant permission to trust in him.

What then? can no remedy, no alleviation be devised? Yes; let us follow the advice of the old Latin poet, and pray for "a sound mind in a sound body," and let us accompany this prayer by such efforts as are suited to the fulfilment. Let all our States, and all our smaller communities adopt the well-known and approved methods of education; and let every species of useful information, practical, scientific, and professional, be as widely disseminated as possible. Let us also remember that, for this life at least, men have *bodies*, as well as *minds*, and that they sympathise so extensively with each other in their growth and development, disease and decay, that whatever measures are adopted for the spread of virtue and intelligence, as well as for the alleviation and cure of disease, should have an adaptation to both a physical and a mental constitution.

Great intellectual advancement might be realized through the same means that we employ for the improvement of the brute animals; but though we may not regulate or restrict the license to increase and multiply, yet one thing may and should be done in this direction. It is a fact as universal as it is lamentable, in this country, that our native, and more attractive and intelligent females are sadly debilitated and degenerated in bodily constitution; and I need not say that this must unavoidably work the deterioration of our whole people—first physiologically, then mentally and morally. Now, a glance at the flood of emigration pouring into this country shows that this evil is artificial and may therefore be exterminated. Our own progenitors, on the other side of the Atlantic, are comparatively exempt from it. Let us but adopt the better portion of their hygienic regimen, especially their custom of daily and prolonged exertion in the open air, and I doubt not but that in a few generations we shall see more healthy mothers and fewer scrofulous children. This reform would be an important auxiliary in the great work of popular education and elevation, the only radical cure, not only for quackery, but also for all our ills—whether physical, mental, social, or moral.

